# Can Central Place Foraging Theory Predict Material Collection Behaviours of the Eurasian Beaver (*Castor fiber*)?

**DOI:** 10.1002/ece3.71325

**Published:** 2025-05-12

**Authors:** Are Værøyvik, Joah R. Madden

**Affiliations:** ^1^ Centre for Research in Animal Behaviour University of Exeter Exeter UK

**Keywords:** beaver, *Castor fiber*, central place foraging theory, construction material, energy maximisation, time minimisation

## Abstract

Central place foragers (CPF) are animals that return collected resources to their central place. During foraging trips, individuals may exhibit different strategies and either prioritize energy maximizing (EM) during foraging bouts or minimize the time (Time minimising, TM) they spend away from their central place. The exhibited strategy may depend on the composition and distribution of the desired resources and costs of local threats. Beavers exhibit CPF, collecting timber for food and for use in constructions. We assessed trees felled by beavers and control, unfelled trees at two rivers in England and Norway that differed in resource availability and distribution and history of beaver occupation. We evaluated evidence to support or refute EM and TM strategies. We found that beavers were selective in the size and species of trees that they felled. However, the beavers differed between sites in whether their felling was spatially clustered or more randomly distributed. These results suggest site‐dependent variation in the predictive value of the EM and TM strategies of CPF for beaver material collection behaviors. These differences between the two sites may reflect different optimal foraging strategies driven by different lengths of occupation by beavers in the vicinity.

## Graphical abstract

We assessed trees gnawed by beavers and control, ungnawed trees at two rivers in England and Norway that differed in resource availability and distribution, predator threat, and history of beaver occupation. Our results provide evidence for site‐dependent variation in the predictive value of the Energy Maximising and Time Minimising strategies of Central Place Foraging for beaver material collection behaviours.

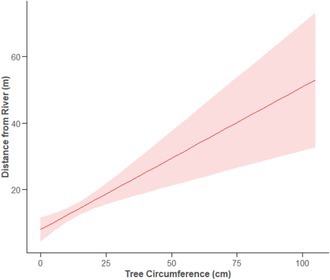

## 1 Introduction

Central Place Foraging (CPF) involves acquiring resources and conveying them to a fixed location, usually central to the distribution of collection points, for example a nest or colony, either for caching or for immediate consumption (Orians and Pearson 1979). Decisions as to what items to acquire and transport from collection points are predicted by Optimal Foraging Theory, whereby the forager attempts to optimize the net gain per unit of energy spent (MacArthur and Pianka 1966). During CPF, where the forager moves the resource away from the collection point before using it (eating it or using it for other purposes at the central place location) the energy costs are highly dependent on the distance from the collection point to the central place, on the physical transport costs of the resource, determined by its mass, bulk, conspicuousness or impediment, and the number of resource items being transported on each trip (Schoener 1979). The forager, carrying a (or several) resource(s) back to their central place, faces both energetic costs of moving the resource and risks of predation due to increased conspicuousness or decreased evasion ability as they travel (Olsson et al. 2008). In order to achieve an optimal resolution about where to collect items from, which particular items to collect, and how many to transport on a given trip, foragers may adopt one of two different strategies.

First, foragers may be “energy maximisers” (EMs) and attempt to optimise their net gain per unit of energy spent (Orians and Pearson 1979; Schoener 1979). This may manifest in foragers transporting larger resource loads from collection points further from the central place, to compensate for the increased energetic costs of the longer journey (Woodgate and Chittka 2018). This may involve the transport of larger single items (where resource size is variable) (e.g., Royama 1970), or transport of more items (where resource size is more uniform) (e.g., Giraldeau and Kramer 1982) as the distance from the collection point back to the central place increases. Second, foragers may be “time minimisers” (TMs), minimising the time (and hence exposure to predators) they spend transporting a fixed amount of energy (Schoener 1974). This may manifest in foragers transporting smaller or fewer items from collection points further from the central place (Schoener 1979), allowing them to travel faster, and so have less time exposed to predators *en route*. The strategy that an individual adopts may depend on the environment that they inhabit, with the spatial distribution of the resources, the transport costs through the environment, and the presence of predatory threats all influencing the adopted strategy (e.g., Burke and Montevecchi 2009; Pierce and Ollason 1987; Pyke 1984).

Beavers (Castoridae) are an unusual example of central place foragers, felling and transporting trees back to a central place where they use them to construct lodges or dams (Rosell and Campbell‐Palmer 2022; Müller‐Schwarze 2011). Most beaver foraging activity is concentrated within the first 10–15 m from the water, typically not extending beyond 60 m, but these distances vary between study sites, dependent on forage availability and the presence of predators (summarised in Rosell and Campbell‐Palmer 2022). Although beavers do fell and transport some timber back to their central place for food (Jenkins 1980; Vorel et al. 2015), they generally strip off bark or use more succulent plants for this (Rosell and Campbell‐Palmer 2022), with the consequence that, unlike many species in which CPF strategies have been studied, in beavers the resources being transported are used for construction rather than consumption. Eurasian beavers *Castor fiber* are currently increasing their range due to artificial (re)introductions in England (Brazier et al. 2020). This presents the opportunity to study the foraging decisions of beavers in habitats where beavers had not acted for centuries and thus where the distribution of slow‐growing resources (trees) is likely to be unaffected by historic felling actions. Their activity in this novel environment can be contrasted with habitats, for example in Norway, where beavers have been active for hundreds of years and already felled and collected trees. These established beaver populations might have altered their environments through their historic foraging, depleting resources or size distributions close to the river bank, altering tree community composition, or changing the spatial distribution of preferred resources, thus altering the conditions for which different foraging strategies might be optimal. Therefore, we ask three questions of beaver foraging behaviour, each of which allows us to make explicit predictions that might support or refute the animals using an EM or TM foraging strategy while comparisons across two sites differing in occupancy duration allow us to consider why any differences in strategies might occur.

First, do beavers show selectivity in which trees they fell, based on their size and location? Timber is heavy, bulky, and awkward to transport on land. Consequently, to both maximise energy gain and minimise time spent, beavers should preferentially collect resources nearer to their central place, or the waterbodies of their territory where transport costs are reduced. Previous findings suggest that this is the case, with the overall level of felling of trees being especially prevalent close to their central place lodge or dam (e.g., Gallant et al. 2004; Haarberg and Rosell 2006). However, it is less clear whether they choose to fell larger or smaller timber as they get further from their central place (e.g., Haarberg and Rosell 2006; Pejstrup et al. 2023). Carrying large trees is likely more costly in terms of time and energy than carrying smaller trees (Jenkins 1980). If beavers apply an Energy Maximising strategy, they likely select larger materials with increasing distance. However, if they apply a Time Maximising strategy, they likely select smaller materials with increasing distance (Schoener 1974).

Second, do beavers show selectivity for certain tree species to fell? If beavers apply an EM strategy to their species selection, they likely show selectivity for species with higher nutritional contents and easier digestibility. Beavers highly prefer poplars (quaking aspen), willows, birches, and hazels (Haarberg and Rosell 2006; Misiukiewicz et al. 2016; O'Connell et al. 2008), which are all nutritious for beavers (Danilov et al. 2011, Rosell and Campbell‐Palmer 2022, 157–159). Quaking aspen and willows are also digested relatively fast (Danilov et al. 2011; Fryxell et al. 1994), which allows for a higher rate of food intake, while coniferous trees have lower digestibility (Fryxell and Doucet 1991) and are generally avoided by beavers (e.g., Donkor and Fryxell 2000; Haarberg and Rosell 2006; Salandre et al. 2017). Alternatively, if beavers apply a TM strategy, they may select species with lower stem toughness to promote quicker felling and less time spent on land. Beavers selected stems of species with lower Janka hardness values for both consumption and use as building materials (Deardorff 2024).

Third, do beavers exhibit clustering in their felling behaviour? Because beavers gather relatively large materials, tree branches, they are often assumed to be single‐item loaders (e.g., Gallant et al. 2004), collecting and bringing back a single item per trip (Woodgate and Chittka 2018). However, beavers are reported to transport bundles of up to seven branches to their central place in a single trip (Rosell and Campbell‐Palmer 2022, 152). If beavers can fell and carry multiple items in a single material collection trip, they would reduce the number of trips required to return the felled materials. This would both maximise energy and minimise time taken in transport. If so, beavers could benefit from identifying clusters of suitable branches, then felling and carrying as many as possible to their central place, resulting in detectable clustering of felling activity. Beavers may also benefit from identifying clusters of preferred materials and revisiting them to reduce the search time or predator exposure of sampling for new collection points (Ferreira et al. 2023). Similar exploitation of clustered resources is seen in other species which prioritise previously successful foraging locations until the resources there are depleted below an acceptable density (e.g., Benedix Jr 1993; Kramer and Weary 1991). Both EM and TM strategies may favour clustered foraging to reduce search time or increase transport efficiency.

The tree felling behaviour of beavers allows researchers to assess their choice behaviour in retrospect. The felled stumps that remain reveal: the species selected; the location of the choice; and the dimensions of the timber that was taken. These measures can be used to test predictions made (above) about how the felling decisions of beavers influence their CPF strategies and whether they may be optimal. We collected the same data from two very different locations in order to probe, and perhaps increase, the generality of any conclusions we might draw. We sampled felled (felled by beaver) and control (not felled, standing) trees at River Otter in Devon, England, and River Gjøv in Åmli, Norway, to assess whether the EM and TM strategies of CPF can predict material collection behaviours of the Eurasian beaver, and if they do so consistently across these two rivers with different ecologies and histories of beaver occupation.

## 2 Methods

### 2.1 Study Sites

**FIGURE 1:**
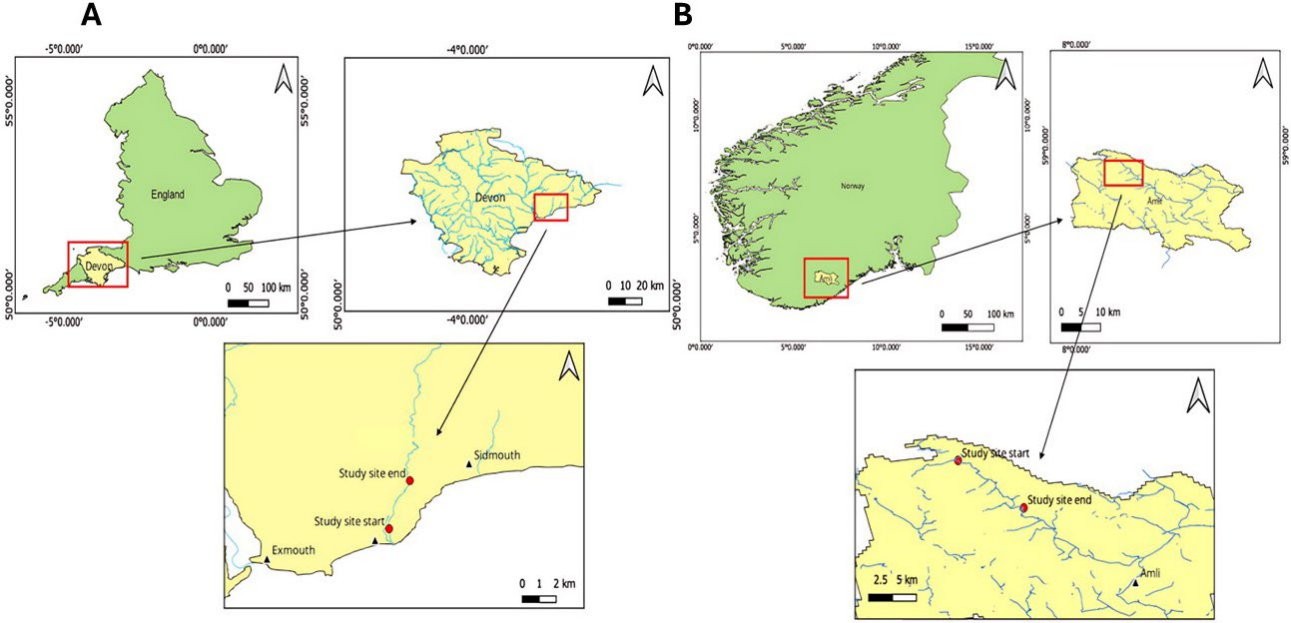
Map of the sites used in this study A) River Otter in Devon, England, and B) River Gjøv in Åmli, Norway.

Data were collected from the River Otter in Devon, England, and River Gjøv in Åmli, Norway (Figure 1). At the River Otter, beavers have recently been reintroduced around 2008, and by 2019, beaver families had established approximately 13 territories along the river and its tributaries (Brazier et al. 2020). We recorded data from the lower main River Otter, from the estuary in Budleigh Salterton (50.6400°N, 3.3098°W), to Colaton Raleigh (50.6745°N, 3.2925°W). The sections of the river used in the present study are characterised by predominantly sandy and grassy riverbanks, dense willow growth limited in reach by a neighbouring fence approximately 5–20 m from the riverbank, generally low variation in riverbank height, and a trail that runs parallel along the river, for the most part within 10 m of the riverbank, popular for walkers and runners and so subject to regular disturbance. There were no dams along the main river, but multiple dams in neighbouring tributaries. The river descended from 17 to 2 m above sea level. The mean river width in the study area was 14.8 m. At the River Gjøv (around Åmli), beavers are long‐established and have been protected by law since 1894 (Richardsen 2012). We recorded data from the upper half of the river, from just after its origin at Lake Gjevden (58.9486°N, 8.1686°W) to Askland (58.8782°N, 8.2876° W). The sections of the river used in the present study are characterised by predominantly rocky and grassy riverbanks, large, dense coniferous forests, larger variation in riverbank height, and less exposure to anthropogenic activities. There is a public county road running along the entire river, but on average 39 m from the riverbank at the study sections (calculated in the same manner as the river width at River Otter). There were no dams along the main river, but they were in neighbouring tributaries. The river descended from 320 to 230 m above sea level. The mean river width in the study area was 25.4 m.

### 2.2 Procedure

We surveyed each river during June and July 2024. Initially, we surveyed the study areas to determine accessibility. This ensured that we could collect data from longer, continuous sections of the rivers. Accessible sections shorter than 75 m were not used, but otherwise, we sampled all accessible sections along the rivers. Due to the width of the river and difficulties of crossing it, at any one section we only surveyed one bank. We surveyed each section twice, once focusing on control trees, and once on felled trees. At both rivers, all felled trees (those that are fully cut such that the upper section has been separated from the stump) in the sections were recorded by walking a series of linear transects parallel to the river bank to a distance of 100 m from the river or a distance when no further felling was observed, whichever was closest. Control trees were selected at random, with different randomisation methods being used at each river, depending on the conditions at each. At the River Otter, the river mostly ran parallel with farm fences. A control tree was selected at every second‐to‐fourth fencepost (roughly every 5–15 m), determined by using a random number generator (RNG) (Dean 2024). The specific tree chosen would stand between the selected fencepost and the nearest point of the river bank and would be determined by two additional samples of the RNG. One sample would decide the distance from the riverbank, divided into three roughly even segments, and another sample of the RNG would determine the specific tree out of the available trees within that segment. There were no fenceposts at River Gjøv. Therefore, to emulate the fencepost strategy, the RNG was used to determine the number of paces (5–15) taken from the current control tree to the next new one along the riverbank, with the next two samples of the RNG being used as described for the River Otter.

For each felled and control tree, we recorded: Site—whether a measurement was made at River Otter or River Gjøv; Type—whether the tree was Felled (visual evidence of beaver felling) or Control (no visual evidence of beaver felling); Species—the vast majority of trees were Willows (*Salix*), Birches (*Betula*), Alders (*Alnus*), Pines (*Pinus*). We could not reliably identify Willows, Birches and Pines to species level so we identified to the level of genus. Species other than these four occurred less than 12 times each (< 1% of the dataset), so we lumped these together and considered them together as Other; Circumference—the circumference of felled trees was measured using a fabric tape (precision 1 mm) just below the cut, and the circumference of control trees was recorded at 30 cm above the ground, with available evidence suggesting mean beaver fell height to typically be between 4 and 50 cm (Wilsson 1971; Skøyen et al. 2018); Distance to River—the distance (in metres) to the nearest point of the riverbank. Coordinates of all trees were recorded using the “MAPS.ME” app on an iPhone. The distance to the nearest point of the riverbank was calculated for each set of coordinate points using the “Shortest line between features” function in QGIS (QGIS development team, 2024). For felled trees only, we collected a measure of clustering, being the number of felled trees or branches within a 2 m radius of the focal felled tree, measured using a tape.

Determining a central place for a foraging beaver is not simple. Although felled timber may be used in dams and lodges, unless individual branches are tracked, it is not possible to know where they are being moved to. Beavers may maintain multiple dams and perhaps several lodges at any one time. The location of dams in particular may change over time as they are damaged by natural or deliberate human intervention. We know that the dams at the Devon site are highly ephemeral, being removed by landowners if they risk causing flooding. Therefore, the central place may vary over short time periods. Because we collected data on historical felling behaviour—the observable stumps—rather than direct observations of felling, we could not attribute specific times to each felling and hence exactly where the nearest lodge or dam that could have been the intended Central Place. Therefore, we used the distance to the river bank as our Central Place because the transport costs (and predation risk) fall markedly once the wood is in the water, such that the main costs of obtaining the timber are likely imposed by distance to the river bank.

### 2.3 Statistical Analysis

To test whether beavers showed selectivity in felling trees based on their size and location, we constructed a binomial model, with a logit link, asking whether the likelihood of being felled differed by distance from the river and/or circumference of trees and whether either of these relationships differed between the two river sites. We initially included a three‐way interaction between all three factors, but this was not significant (*p* values obtained using the drop1 function) and did not improve model fit (evaluated using AIC), so we dropped it but retained all main effects and two‐way interactions. Full model structures can be seen in the accompanying R script. Diagnostic plots were used to confirm model assumptions were met. We used generalized linear models with a Gamma distribution and log link (because the distance and circumference measures were strongly right skewed) to explore differences in (a) distances from felled and control trees to the river between the two sites and; (b) circumferences of felled and control trees between the two sites, testing if circumference or distance was related to whether the tree was felled or a control, or the river site, with a two‐way interaction between site and felled (or not) allowing us to consider consistency across sites. Some distance measures were zero—being in the river—so we added a small value (0.1) to all values to conform to assumptions of gamma distributions. Models were fitted in R (R core team, 2021). To test whether beavers showed preferences for particular tree species, we used Chi‐square tests to determine whether the felled trees were a non‐random subset of the available control trees. A separate analysis was conducted for each river because the community composition at the two rivers was markedly different. To test whether beavers expressed a preference for clustered items, we calculated the mean and variances of the number of felled items within a 2 m radius at each location point and determined the variance‐to‐mean ratio. We used this approach rather than a Point Pattern Analysis because the trees were more approximately distributed in one dimension, a linear row along the riverbank, rather than in two dimensions (covering an area of land) for which a PPA might be more appropriate. This was conducted separately for the two rivers. Chi‐square tests and clustering analyses were conducted by hand.

### 2.4 Ethical Considerations

The project was approved by the University of Exeter Psychology Ethics Committee (approval number #5398090). To ensure minimal disruption to the habitats and beavers, the study areas and their respective sections where data collection took place were only visited once, during the day when beaver activity levels were naturally lowest.

## 3 Results

### 3.1 Do Beavers Show Selectivity in Which Trees They Fell, Based on Their Size and Location?

**FIGURE 2:**
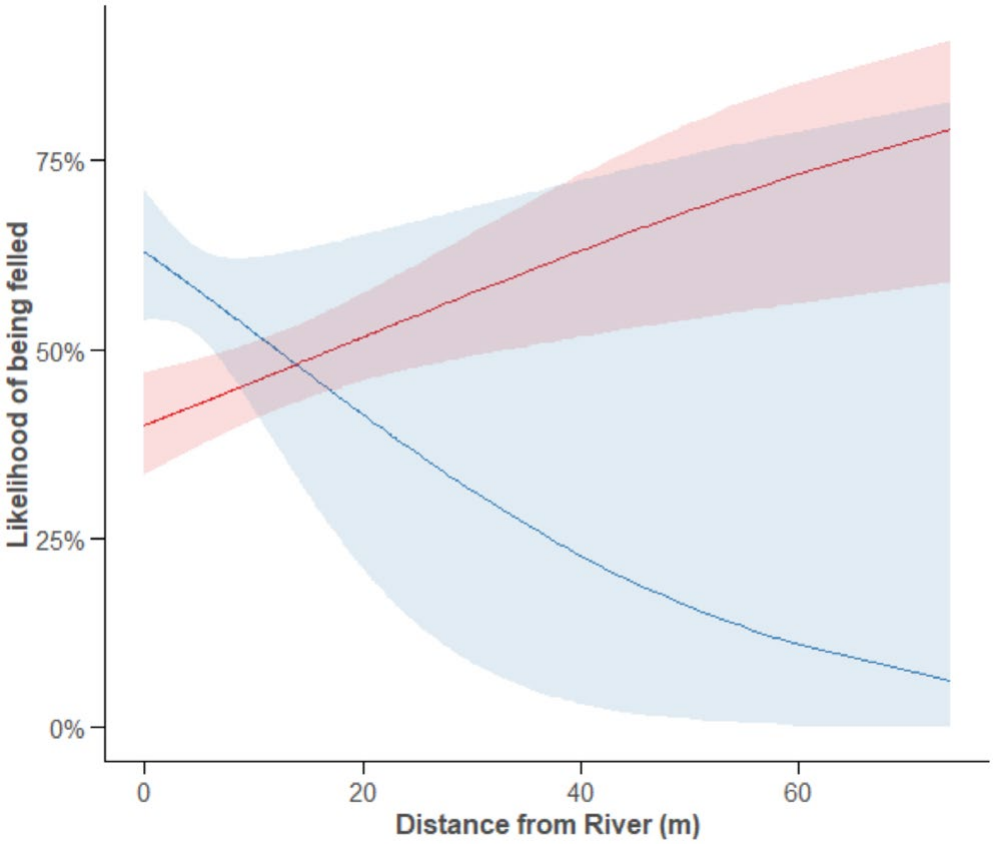
The likelihood of a tree being felled depending on its distance from the riverbank for the two rivers (River Gjøv = Red, River Otter = Blue). Shaded areas indicate 95% CIs.

**FIGURE 3:**
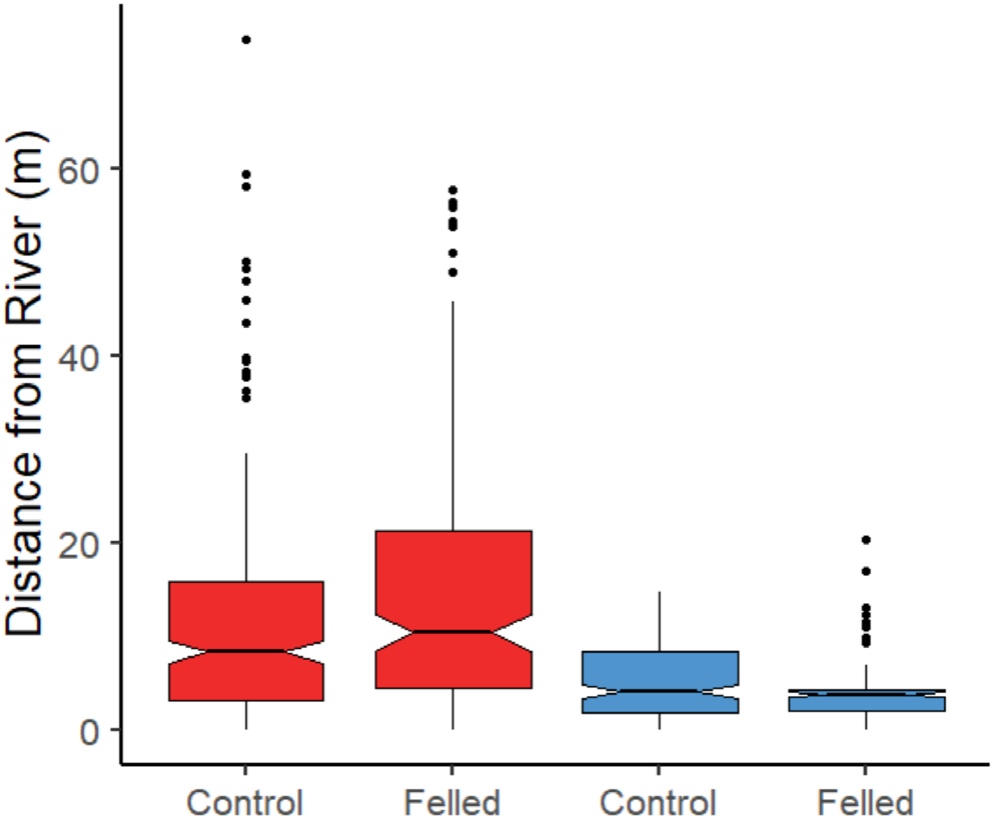
Distributions of the mean tree distance from the riverbank for felled and control trees between the two rivers (River Gjøv = Red, River Otter = Blue). The black bar in each denotes the median, the bottom and top of each box denote the first and third quartile of the data, the whiskers denote the highest and lowest value at most 1.5*IQR from the quartiles. Outlying points are shown individuals as black dots. The notches give a roughly 95% CI for comparing the medians.

The probability of a beaver felling a tree depending on its distance from the river bank differed between sites (Distance × River: *χ* ^2^ = 4.31, *p* = 0.038, Figure 2). At the River Otter, a tree was more likely to be felled if it was close to the river, and more unlikely to be felled further away, accounting for the availability of trees at those distances. By contrast, at the River Gjøv, trees were more likely to be felled when further (up to 60 m) from the river and less likely to be felled when close to the river, again, accounting for the availability of trees at those distances. Although all trees were on average closer to the river bank at the River Otter than at River Gjøv (Table 1), at River Gjøv, felled trees (Median (*M*) = 10.5 m, IQR = 17.0) were further from the river than control trees (*M* = 8.4 m, IQR = 12.7), while at River Otter, felled trees (*M* = 3.9 m, IQR = 2.2) were closer to the river than control trees (*M* = 4.15 m, IQR = 6.55), suggesting that beavers at River Gjøv selected trees further from the riverbank relative to what was available, while beavers at River Otter selected trees closer to the riverbank relative to what was available (Table 1, Figure 3).

**TABLE 1:** Output from linear model of the relationship between the distance of the tree to the river and whether it was felled or not, site, and the interaction between whether it was felled or not and site.

Distance	*b* ± s.e.	*t*	df	*p*
River	−0.79 ± 0.087	−9.14	3, 838	< 0.001
Type (Felled vs. Control)	0.33 ± 0.085	−4.29	3, 838	< 0.001
River × Type	−0.55 ± 0.13	−4.29	3, 838	< 0.001

**FIGURE 4:**
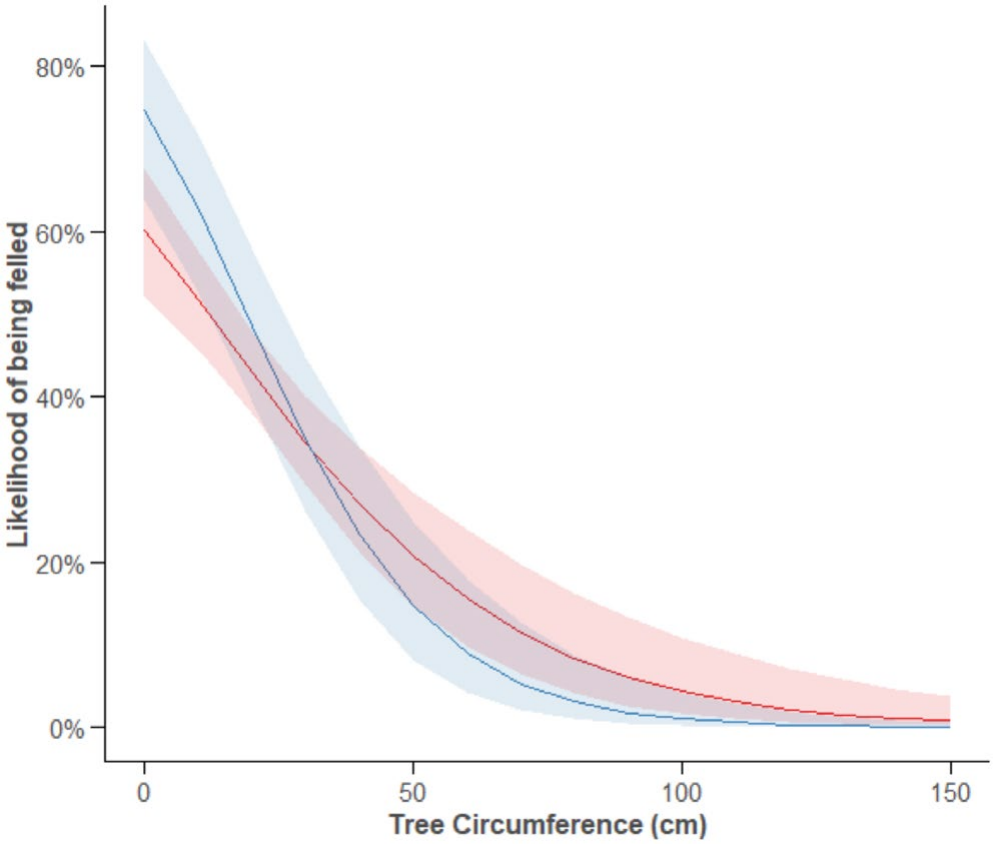
The likelihood of a tree being felled depending on its circumference for the two rivers (River Gjøv = Red, River Otter = Blue). Shaded areas indicate 95% CIs.

**FIGURE 5:**
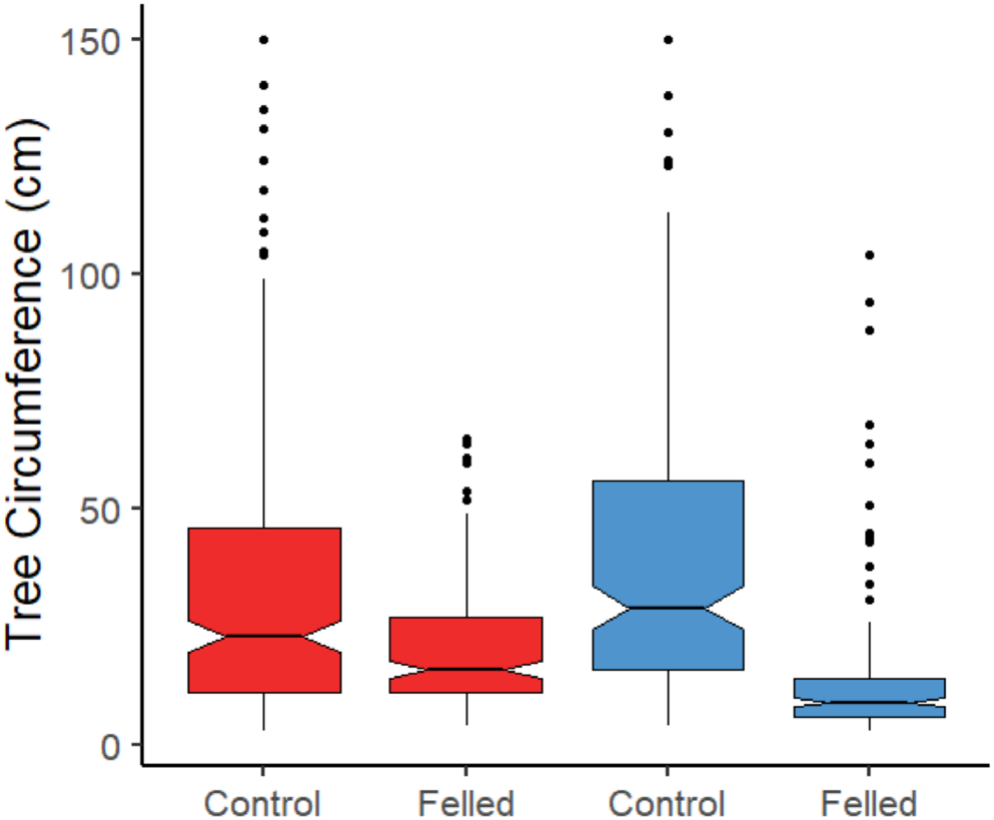
Distributions of the circumference of felled and control trees between the two rivers (River Gjøv = Red, River Otter = Blue). The black bar in each denotes the median, the bottom and top of each box denote the first and third quartile of the data, the whiskers denote the highest and lowest value at most 1.5*IQR from the quartiles. Outlying points are shown individuals as black dots. The notches give a roughly 95% CI for comparing the medians.

There was also a tendency for the probability of a beaver felling a tree depending on its circumference differing between sites (Circumference × River: *χ* ^2^ = 3.66, *p* = 0.056, Figure 4). Overall, smaller trees were more likely to be felled, but smaller trees were proportionately more likely to be felled and larger trees less likely to be felled at the River Otter, compared to River Gjøv. On the River Otter, the felled trees were 44% smaller (Median (*M*) = 9 cm, IQR = 8) than those felled on the River Gjøv (*M* = 16 cm, IQR = 16) and 69% smaller than those trees available but unfelled (*M* = 29 cm, IQR = 40), even though the unfelled, available trees at this site were 26% larger than those on the River Gjøv (*M* = 23 cm, IQR = 35.2) (Table 2, Figure 5).

**TABLE 2:** Output from the model of the relationship between tree circumference and whether it was felled or not, site, and the interaction between whether it was felled or not and site.

Circumference	*b* ± s.e.	*t*	df	*p*
River	0.17 ± 0.088	1.99	3, 861	0.047
Type (Felled vs. Control)	−0.63 ± 0.085	−7.42	3, 861	< 0.001
River × Type	−0.57 ± 0.13	−4.49	3, 861	< 0.001

**FIGURE 6:**
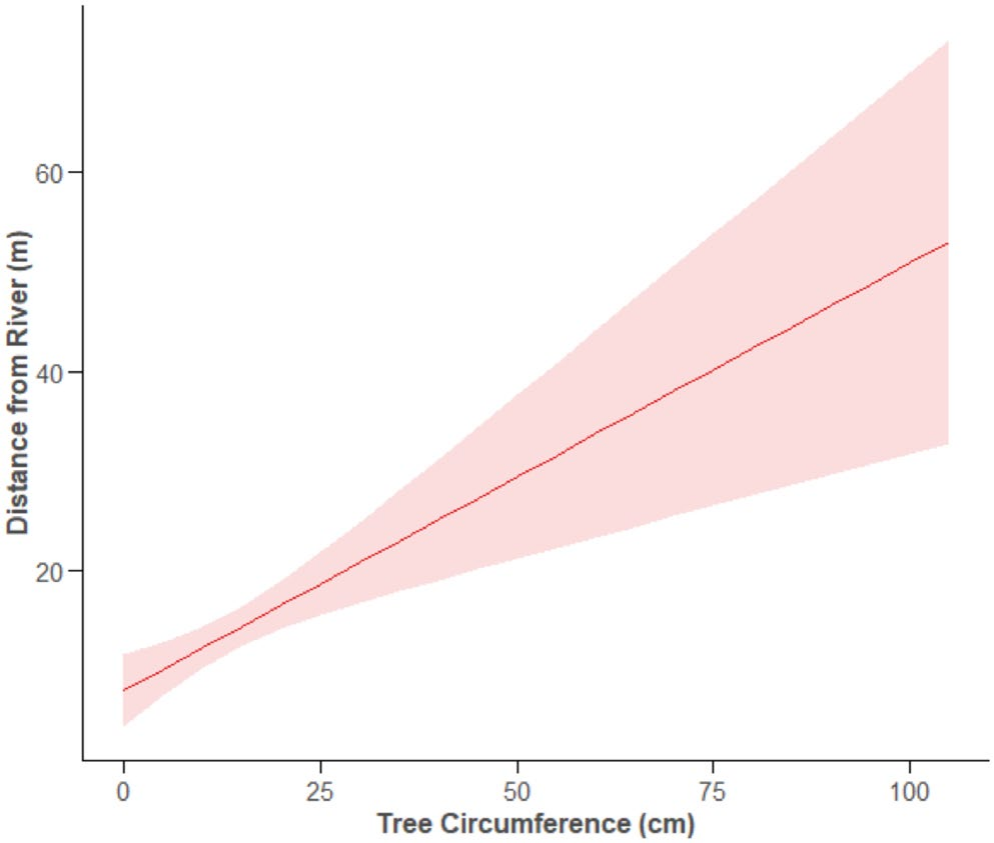
The relationship between the circumference of a tree that was felled and its distance from the river for felled trees Shaded areas indicate 95% CIs.

For the trees that were felled, we found that beavers felled larger trees when further from the river, and this relationship was consistent across the two rivers (Table 3, Figure 6).

**TABLE 3:** Output from model of the relationship for felled trees between distance to the river and circumference, site, and the interaction between distance and site.

Distance	*b* ± s.e.	*t*	df	*p*
Circumference	0.42 ± 0.12	3.82	3, 378	< 0.001
River	−6.19 ± 1.88	−3.29	3, 378	0.0011
Circumference × River	−0.23 ± 0.12	−1.87	3, 378	0.062

### 3.2 Did Beavers Show Selectivity for Certain Tree Species to Fell?

**FIGURE 7:**
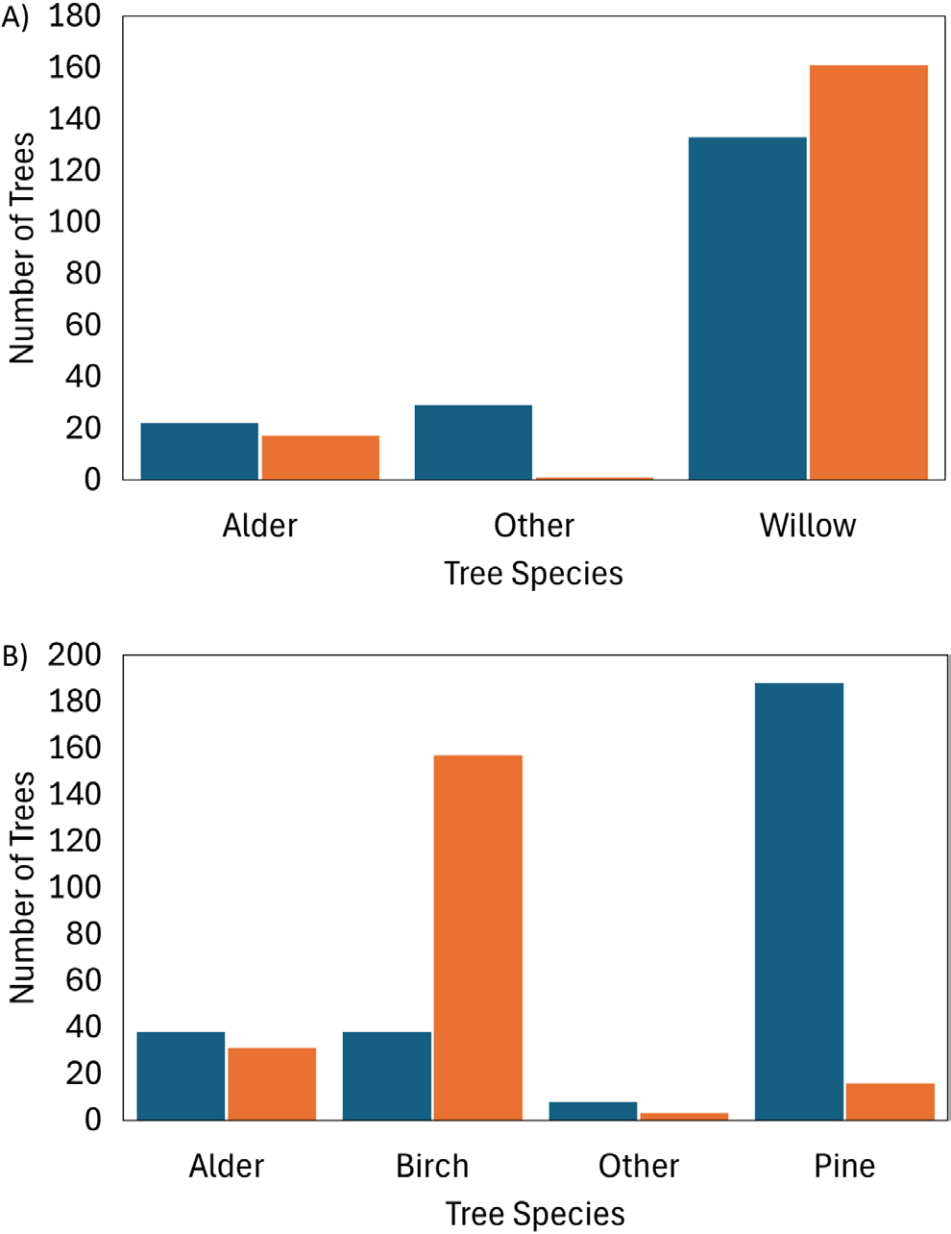
The number of trees of each species recorded at (A) River Otter and (B) River Gjøv that were felled (Orange bars) or unfelled (Blue bars).

Beavers at River Otter did not select each species in proportion to its availability (*χ* ^2^_1_ = 29.38, *p* = < 0.001). More Willow trees and fewer Other trees than expected were felled (Figure 7A). Beavers at River Gjøv also did not select each species in proportion to its availability (*χ* ^2^_1_ = 218.35, *p* = < 0.001). More Birch, but fewer Pine trees were felled than expected given their availability (Figure 7B). At both rivers, Alder trees were felled about as much as expected given their availability (Figure 7).

### 3.3 Did Beavers Exhibit Clustering in Their Felling Behaviours?

Each felled tree at the River Otter was in close proximity (< 2 m) to a mean of 4.96 other felled trees. They were isolated (no other felled trees within 2 m of a focal felled tree) at 38% of locations. The variance‐to‐mean ratio of the number of felled trees per site was 9.12, considerably higher than 1, thus indicative of clustering. Each felled tree at the River Gjøv was in close proximity to a mean of 2.20 other felled trees. They were isolated at 50% of locations. The variance‐to‐mean ratio of the number of felled trees per site was 1.30, approximately equal to 1, indicative of a random distribution of felling.

## 4 Discussion

We observed the felling behaviour of beavers, indicating their collection of stick material for food and constructions, at two sites. We found general patterns in their felling patterns across sites depending on the size and species of trees. At both sites, beavers were selective about which species they felled, preferring some species (Birch in Norway, Willow in Devon) while avoiding others (Pine in Norway and Others in Devon). They were also generally selective in the size of trees that they felled, preferring smaller trees over larger ones available in the area, with this preference pattern being stronger in Devon than in Norway. However, we found marked differences in their felling behaviours between the sites, depending on the location of the trees. In Devon, a tree was less likely to be felled the further it was from the river, but in Norway, trees further away from the river (up to 60 m) were more likely to be felled. If a tree was felled, then, in Norway, the further it was from the river, the larger it was, but in Devon, there was no such relationship. Further, in Devon, beavers were more likely to fell multiple trees in a local cluster, whereas in Norway, we found no evidence of this clustering, with instead a more random pattern of felling. These differences between sites may reflect different optimal foraging strategies, driven by different lengths of occupation by beavers in the vicinity. If these results are extended across multiple sites, then they could provide evidence for site‐dependent variation in the predictive value of the EM and TM strategies of CPFT for beaver material collection behaviours. We acknowledge that EM and TM strategies are not always mutually exclusive, and their manifestation may be dependent on tree availability, distribution, and/or quality. Individual beavers or populations may operate mixed strategies, with the emphasis on EM or TM depending on contextual factors such as patch quality, central place distance, or risk exposure. We also acknowledge that our use of the riverbank as the central place, rather than the lodge or dam, may explain differences in our findings from those of other studies. However, the gross differences between the two sites in felling behaviours that we detected at them indicate that the beavers living there are operating differing choice strategies.

On the River Otter, the preference for material collection nearer the riverbank compared to what was available is in compliance with CPFT and supports both EM and TM strategies. It is surprising that beavers at River Gjøv preferred materials further from the riverbank compared to what was available. It opposes previous findings (e.g., Gallant et al. 2004; Haarberg and Rosell 2006), and what would be predicted by the EM and TM strategies of CPFT. The increased energetic costs and predation risk of foraging at greater distances make this a purely disadvantageous action when considered on its own. Therefore, it is likely that this preference was produced by another factor. One potential example of such a factor is the lack of availability of preferred materials closer to the river. Coniferous trees, which are strongly selected against by beavers (e.g., Donkor and Fryxell 2000; Gallant et al. 2004), accounted for 68% of the recorded control trees at River Gjøv. This suggests that the majority of the available tree species in the survey area close to the river were unfavorable. Additionally, their most preferred species, birches, were strongly selected for, and occurred much less frequently as control trees (Figure 7B). This could indicate that beavers at River Gjøv have already exploited the majority of the available birch trees over their long period of occupancy and must journey further to find remaining ones. This seems sensible given the contrasting findings at River Otter, where the most frequently felled species, those within the willow genus, were also the most frequently occurring control trees (Figure 7A) suggesting that the recently established beaver populations there have yet to deplete the preferred resource.

At both rivers, beavers selected larger material sizes with increasing distance from the riverbank, matching several previous studies (e.g., Fryxell and Doucet 1991; Gallant et al. 2004; Haarberg and Rosell 2006; McGinley and Whitham 1985; Raffel et al. 2009). This suggests that beavers apply an EM strategy when deciding which sizes to fell as distance increases and does not support the prediction that beavers exhibit a TM strategy and select smaller materials with increasing distance due to the relatively large materials they gather (Jenkins 1980), as a result of the increased costs large load sizes should induce (Olsson et al. 2008). Felling larger trees does not have to mean carrying larger trees, as beavers sometimes strip stems of their branches, leaving the main stem behind Wilsson (1971) cited in Rosell and Campbell‐Palmer (2022). They may also eat the bark of such stems at the harvest site (Rosell and Campbell‐Palmer 2022, 156). If beavers can obtain more bark or branches from larger trees than from smaller trees, then a positive size‐distance relationship seems reasonable. However, several other studies reveal that beavers select smaller items with increasing distance from the riverbank (e.g., Belovsky 1984; Jenkins 1980; Pinkowski 1983; Raffel and Gatz 2003; Salandre et al. 2017). Salandre et al. (2017) suggested that the difference between studies reporting a negative size‐distance relationship and those reporting a positive size‐distance relationship is that those reporting the latter featured predominantly smaller materials. However, our study featured size ranges and mean sizes larger than or comparable to all cited studies reporting a negative size‐distance relationship, suggesting that this is not the case. It is possible that ecological differences are responsible instead. Our study compared two rivers that differed in several ecological aspects, such as nearby forest density, species availability, elevation range, river width, and possibly degree of anthropogenic disturbance. Differences may also arise depending on the destination of the felled material—whether it is used in dams or lodges—and future studies that track where felled material is taken to might help clarify this. Despite these differences, a positive size‐distance relationship was found at both rivers. Further work at a wider variety of sites is required to explore these potential explanatory factors.

Beavers at River Otter exhibited clustering in their felling locations, supporting both CPFT strategies of EM and TM. Such clustering suggests that beavers at River Otter prioritize previously visited locations with high densities of suitable materials (e.g., Benedix Jr 1993; Kramer and Weary 1991), and/or fell and carry bundles of materials back to their central place (Rosell and Campbell‐Palmer 2022, 152). In contrast, beavers at River Gjøv did not exhibit felling clusters, and so did not support predictions of the EM and TM strategies of CPFT. This difference may have been caused by the lack of available clustered materials at River Gjøv. River Otter had stands of rather small willows growing along much of the riverbank. In contrast, the birches at River Gjøv were often larger, single trees. The, on average, larger single trees felled at River Gjøv might also be only carried individually rather than in bundles, reducing the value of clustered felling. Our metric of clustering was fairly crude and future studies that more accurately mapped the density and spatial positioning of all trees at a site and then concurrently tracked beaver movements through the landscape as they sampled and exploited clusters could reinforce our findings.

Beavers at River Otter preferentially felled the nutritionally rich willow trees, more than expected given their availability. Alder trees were selected in proportion to their availability, suggesting that they were not strongly selected for. “Other” tree species were never selected, suggesting that beavers at River Otter predominantly collect willows and alders. The selectivity of willows is predicted by CPFT as an EM strategy due to its high nutritional content and efficient digestibility (Danilov et al. 2011, Rosell and Campbell‐Palmer 2022, 157–159). However, willows also have low Janka hardness values (Meier 2015), which means that they could also be selected following a TM strategy. The absence of available species with higher Janka hardness values at River Otter makes it hard to draw conclusions about which CPFT strategy is most applicable. At River Gjøv, beavers predominantly collected materials from the nutritionally rich birch trees and selected them disproportionately often compared to their availability. Alder trees were selected in proportion to their availability, suggesting that they were not strongly selected for. Pine trees were strongly avoided, matching previous literature (Donkor and Fryxell 2000; Haarberg and Rosell 2006; Salandre et al. 2017). Given the high Janka hardness values of the preferred, nutritionally rich birches and the low Janka hardness values of the avoided, less efficiently digested conifers, it seems that beavers at River Gjøv act in accordance with an EM strategy in their species selection. A set of study sites where a wider mix of tree species differing in hardness and nutritional value would be needed to confirm the importance of these factors in shaping felling choice in conjunction with other ecological factors.

We suggest that there is site‐dependent variation in the predictive value of the EM and TM strategies of CPFT for beaver material collection behaviours in preferences for foraging distance and clustered materials at two sites in England and Norway. The differences may have been caused by resource availability arising from the different histories of beaver occupation at the two sites. For example, long‐term occupation by beavers might have depleted resources or size distributions close to the river bank, altered tree community composition in the area, or led to changes in clustering of the spatial distribution of preferred resources. However, to confirm this, additional studies at a range of other sites that differ in their histories of occupation and natural resource distribution are desirable. The differences that we observed may also arise because the underlying environmental conditions of the sites naturally differ, due to soil types, nutrient levels, or climate. A more targeted survey approach would allow these potential confounds to be disentangled. As new beaver populations in the UK spread and become established, sites with a greater spread of occupancy histories yet sharing more common underlying environmental conditions may become available in the coming decades. We found that the EM strategy predicted the behaviours of beavers at both rivers in their selection of material size with increasing distance and likely in their species selection. The size‐distance relationship findings are contested across the literature and may be influenced by local environmental, population, or individual differences. Consequently, studies assessing foraging/material collection behaviours of CPFs should be careful in making species‐general conclusions from their findings without a clear understanding of how a focal species might behave in environments with different histories of occupation, resource composition, and distribution.

## Author Contributions

**Are Værøyvik:** conceptualization (equal), formal analysis (equal), investigation (lead), methodology (equal), writing – original draft (lead), writing – review and editing (supporting). **Joah R. Madden:** conceptualization (equal), formal analysis (equal), methodology (equal), writing – original draft (supporting), writing – review and editing (lead).

## Conflicts of Interest

The authors declare no conflicts of interest.

## Supporting information

[THIS CAN BE REMOVED. THE CODE IS NOW HOSTED ON DRYAD]

## Data Availability

Data and code are available on Dryad https://doi.org/10.5061/dryad.h18931zxx For the purpose of open access, the author has applied a Creative Commons Attribution (CC BY) licence to any Author Accepted Manuscript version arising from this submission.
